# Kidney Transplantation in Patients With Active SARS-CoV-2 Replication: An Initial Case Series

**DOI:** 10.3389/ti.2022.10716

**Published:** 2022-08-25

**Authors:** Matthieu Halfon, Louis Stavart, Jean-Pierre Venetz, Oriol Manuel, Dela Golshayan

**Affiliations:** ^1^ Transplantation Center, Lausanne University Hospital, University of Lausanne, Lausanne, Switzerland; ^2^ Service of Infectious Diseases, Lausanne University Hospital, University of Lausanne, Lausanne, Switzerland

**Keywords:** SARS-CoV-2, kidney transplantation, vaccination, immunosuppression, monoclonal antibodies, induction therapy

Dear Editors,

Since 2020, the severe acute respiratory syndrome coronavirus (SARS-CoV-2) pandemic has had a negative impact on transplant recipients and on many transplantation programs. With the persisting high prevalence of infections in the general population, transplant nephrologists will be facing patients with positive swabs at the time of organ allocation and transplantation. Patients with end-stage renal disease (ESRD) and kidney transplant (KTx) recipients have low serologic responses to vaccination and are at high risk of severe COVID-19 (1). In the immediate post-transplant period, patients are under a high burden of immunosuppressive therapies, including induction regimens based on anti-lymphocytes antibodies, to prevent acute rejection and early immunization. Current international guidelines generally recommend to wait for at least 2 weeks after acute infection and to have a negative detection of SARS-CoV-2 RNA in a nasopharyngeal swab before moving forward to transplantation (2). It is also highly suggested to postpone transplantation in case of suspected or confirmed SARS-CoV-2 infection (2). However, with the advent of the omicron variant, associated with lower severity, it is not known whether transplantation is safe in patients within shorter time from infection and with detectable RNA in the nasopharyngeal swab (3). We present our recent cases of KTx performed in SARS-CoV-2 positive patients.

Our institution is a tertiary hospital in Switzerland, performing approximately 60 KTx per year with around 50% living donors. Since the beginning of the pandemic, all patients called in hospital following an organ offer undergo a nasopharyngeal swab, as part of the routine tests performed before transplantation. Between January, 1st and March, 31st 2022, four patients with significantly detectable SARS-CoV-2 RNA in a nasopharyngeal swab at the time of surgery underwent KTx. Three out of four organs came from deceased donors. For the patient with a living donor, the planned transplantation was previously postponed due to the shutdown of elective surgical activities in our institution at the peak of the pandemic. Baseline characteristics of the patients and their outcome are detailed in Table 1. Three patients had an mRNA-based SARS-CoV-2 vaccination history. The decision to transplant was taken regardless of post-vaccination serology values, as these results were not rapidly available. Viral genotyping was not done systematically in our center, but all transplantations occurred during the peak of the omicron wave in Switzerland, and in particular of the initial BA.1 sublineage (>90% of SARS-CoV-2 infections, as monitored by the Swiss Federal Office of Public Health) (4). The mean time between the diagnosis of infection and transplantation was 13 (range 5–16) days. All patients were paucisymptomatic (slight rhinitis or dysphagia) at the time of surgery. After transplantation, the recipients were carefully monitored clinically, together with daily blood tests and SARS-CoV-2 nasal swabs every 48 h (until a negative PCR). In 2 out of 4 patients, repeated nasal swabs showed still increasing viral titers in the first 48 h, but for all patients the tests became negative by day 10 after transplantation with full resolution of the initial mild symptoms. Induction regimen was basiliximab for all recipients, together with high dose corticosteroids (methylprednisolone pulses during the first days at tapering doses followed by prednisolone 20 mg/day from day 5 onwards), tacrolimus and mycophenolate mofetil. One patient with preformed donor-specific anti-HLA antibodies (DSA) received in addition intravenous immunoglobulins (IVIG, 2 g/kg). At 1 month, no patient had presented symptomatic COVID-19 or other related infections. None of the patients developed an episode of acute rejection or detectable anti-HLA antibodies in the first 3 months of follow-up, with excellent and stable kidney function at 1 and 3 months. Thus, in our small case series, a positive nasopharyngeal swab at the time of transplantation was not associated with COVID-19 disease progression or other unfavorable post-transplant outcomes.

**TABLE 1:** Patients’ characteristics at transplantation and outcome.

Patient	1	2	3	4
Age at Tx (y), gender	53, male	30, female	38, male	54, male
Nephropathy	Hypertensive nephropathy	Lupus nephritis	IgA nephropathy	Diabetic nephropathy
First Tx	Yes	Yes	Yes	Yes
HLA immunization status	Preformed class II DSA	No DSA	No DSA	No DSA
Preemptive Tx	No, HD for 2.3 years; since 4 months on the waiting list	Yes	Yes	No, HD for 3.5 years; since 3 years on the waiting list
Diabetes	No	No	No	Yes
Cardiovascular comorbidities	No	No	No	Yes
Days between SARS-CoV-2 infection^a^ and Tx surgery	15	16	16	5
Vaccination status at the time of Tx	3 injections of mRNA vaccine, last injection 18 days before Tx	3 injections of mRNA vaccine, last injection 100 days before Tx	Not vaccinated before Tx	2 injections of mRNA vaccine, last injection 24 days before Tx
Viral load by PCR (nasal swabs) at the time of Tx	7200 copies/ml (pic value at 46,000 copies/ml, at day 6)	36,000 copies/ml (pic value at 89,000 copies/ml, at day 2)	3500 copies/ml (highest value)	150,000 copies/ml (highest value)
Induction immunosuppressive regimen	Bas/CS/TAC/MMF and IVIG	Bas/CS/TAC/MMF	Bas/CS/TAC/MMF	Bas/CS/TAC/MMF
Sotrovimab injection	Yes, 15 days before Tx	Yes, 1 day after Tx	Yes, 1 day after Tx	Yes, 3 days before Tx
Outcome at 1 month	No COVID-19-related symptoms or complications	No COVID-19-related symptoms or complications	No COVID-19-related symptoms or complications	No COVID-19-related symptoms or complications
	Negative nasal swab PCR 8 days after Tx	Negative nasal swab PCR 10 days after Tx	Negative nasal swab PCR 4 days after Tx	Negative nasal swab PCR 9 days after Tx
Kidney function after Tx (serum creatinine)	133 and 128 µmol/L, at 1 and 3 months, respectively	69 and 73 µmol/L, at 1 and 3 months, respectively	135 and 127 µmol/L, at 1 and 3 months, respectively	81 and 78 µmol/L, at 1 and 3 months, respectively

a As per patients’ history and available PCR tests performed in the dialysis centers before Tx.

Bas, basiliximab; CS, corticosteroids; DSA, donor-specific anti-HLA antibodies; HD, hemodialysis; IVIG, intravenous immunoglobulins; MMF, mycophenolate mofetil; TAC, tacrolimus; Tx, transplantation; y, years.

The cumulative dose of immunosuppression received during the induction period and early months after transplantation is well described to put the patients at high risk of infections. However, the risk of undergoing KTx with acute SARS-CoV-2 infection is still unknown and could expose those recipients to severe complications. So far, immunocompromised patients, such as patients with autoimmunity or after transplantation, were shown to have higher rates of severe disease progression after SARS-CoV-2 infection compared to the general population (5). Thus, facing the decision to perform an elective KTx, clinicians have to balance the risk of worsening of COVID-19 in a paucisymptomatic recipient, versus declining the organ and possibly an excellent transplantation opportunity in particular in immunized recipients. However, compared to the delta variant, current data suggest that omicron has a reduced severity, particularly in vaccinated patients.

The availability of antiviral as well as monoclonal antibodies (mAbs) against SARS-COV-2 may offer new possibilities to allow the transplantation of patients in a context where the virus is still endemic. At least for low-immunological risk recipients, the procedure should be safe providing the use of induction protocols without lymphocytes-depleting therapies. Indeed, T cells play a major role in the immune response against viruses, including SARS-CoV-2 (6). Administration of anti-thymocyte globulins was associated with more complications and more severe COVID-19, compared to basiliximab-based induction in transplant recipients (7). B cells and the antibody response against SARS-CoV-2 are also important to prevent severe disease. The B-cell depleting mAb rituximab is widely used in patients with DSA and is associated with three times more risk to develop severe COVID-19 and longer hospital stays (8). Thus, high-immunological risk patients appear to have few safe options for induction therapies. In our small series, three out of four patients were at low-immunological risk, and one patient with preformed DSA could be successfully transplanted with basiliximab induction combined with IVIG, without early acute rejection. Because dialysis patients are known to have lower titers of protective antibodies after SARS-CoV-2 vaccination (9), sotrovimab was administered in all our recipients following the positive PCR test.

In conclusion, an induction protocol based on a combination of basiliximab with mAbs against SARS-CoV-2 Spike protein (sotrovimab at the time of our study) seems to be safe and effective to prevent symptomatic disease and complications in newly transplanted ESRD patients infected with the virus, at least in the context of paucisymptomatic infections and/or low viral loads. With the persisting high prevalence of SARS-CoV-2 in the general population, our preliminary findings are important for KTx programs. If the safety is confirmed in bigger series, the transplantation activity could be maintained despite the pandemic, in particular for patients that have been on the waiting list for a long time, such as the immunized recipients for whom suitable organs are scarce.

## Data Availability

The original contributions presented in the study are included in the article/supplementary material, further inquiries can be directed to the corresponding author.
